# Post-Pandemic Multidimensional Integration of Afghan Refugee Women in the U.S.

**DOI:** 10.1007/s10903-025-01827-2

**Published:** 2025-11-29

**Authors:** Atefeh Aghaei, Shan Qiao, Ben Roth, Patrick Koga, Xiaoming Li

**Affiliations:** 1 University of South Carolina, Columbia, USA; 2 University of California, Davis, Davis, USA

**Keywords:** Afghan refugee women, Refugee integration, COVID-19, Cultural competence

## Abstract

Amid the recent global refugee crisis, the United States (U.S.) hosts over 200,000 Afghan refugees, many struggling to integrate into U.S. society. The COVID-19 pandemic and its subsequent health and social sequelae have severely affected Afghan refugees’ lives, especially women, further complicating their integration. This study examines how the pandemic has influenced Afghan refugee women’s integration through challenges and facilitators at various socio-ecological levels. Semi-structured interviews with 34 Afghan refugee women and 18 service providers in California were conducted. Interview data were analyzed using the thematic framework to identify themes indicating the impacts of COVID-19 on refugee women’s integration based on Ager and Stange’s integration framework. Findings revealed multifaceted challenges in refugee women’s integration across multiple socio-ecological levels exacerbated by the pandemic including changes in roles, financial illiteracy, language barriers, cultural adaptation issues, inefficient policies, patriarchal culture, administrative overload, and systemic barriers in employment and education. However, factors like empowerment through education and employment, family/friends/community support, socializing, childcare support, awareness of laws and women’s rights, and access to culturally competent job opportunities facilitated their integration. This study recommends policies and programs addressing Afghan refugee women’s integration challenges and facilitators impacted by the pandemic for better integration into the U.S.

## Background

The current refugee crisis has led to over 6.4 million Afghan refugees and asylum representing one of the largest refugee populations globally [1]. More than 200,000 Afghans have resettled in the United States (U.S.) during the last two decades, many after the 2021 Taliban takeover [2]. The COVID-19 pandemic and its subsequent biological, mental, and social sequelae, have severely impacted refugees’ lives in the U.S. including Afghan refugee women [3, 4]. While the COVID-19 pandemic’s short-term effects on Afghan refugee women’s health were significant [5], its potential long-term damage to their integration could be devastating. Yet, knowledge about how Afghan refugee women integrate into U.S. society and COVID-19’s impact on this remains limited.

Integration into the host society is key for refugees, seen as their journey to becoming recognized members of the host society [6]. Refugee integration involves multiple dimensions: social, economic, legal, cultural, and political [6, 7]. Unlike other migrants, refugees face unique integration hurdles, including involuntary relocation and structural barriers like discrimination and residency uncertainties in host countries [8, 9]. Existing theoretical models often study refugee integration by looking at individual and community traits affecting their adaptation [10]. For example, past research has shown that personal factors like gender and parenthood significantly impact key domains of refugee integration such as language learning, employment, education, and cultural norms [11, 12].

The COVID-19 pandemic has intensified existing inequalities, making access to healthcare, education, legal protection, and economic opportunities more challenging for refugees [3, 4]. The interruption of essential supplies, such as food and medicine, and restrictions on movement and social distancing measures further compounded these hardships. The pandemic intensified financial and legal barriers which affected refugees’ ability to secure employment and access to social services, crucial for achieving financial independence and security. Heightened social isolation, unemployment, and challenges in accessing accurate health information were other impacts of COVID-19 on refugees [3, 4]. Moreover, COVID-19 has intensified violence and insecurity against refugees, especially refugee women [13, 14]. The closures of state and local administrations during the pandemic have also prevented some refugees from obtaining or renewing their permits for residency or work [15]. The mental health impact of the pandemic on refugees was also significant, with increased reports of anxiety, depression, and post-traumatic stress [16].

Despite much knowledge on COVID-19's impact, research on how the pandemic has affected Afghan refugee women’s integration into the U.S. remains unexplored. In this study, we explored the experiences of Afghan refugee women in the U.S. during the COVID-19 pandemic through semi-structured interviews with them and their service providers recruited from California, U.S. We analyzed interviews to explore the impact of COVID-19 on Afghan refugee women’s integration, focusing on challenges, facilitators, and mental health using Ager and Strange’s framework. This study addresses three key research questions: (1) What are the facilitators and challenges of integration into U.S. society for Afghan refugee women at different socio-ecological levels? (2) How has the COVID-19 pandemic impacted Afghan refugee women’s integration into U.S. society? (3) How has altered integration of Afghan refugee women during the pandemic impacted their mental health?

## Theoretical/Conceptual Framework

While no standard integration framework is accepted by all scholars [9, 17, 18], Ager and Strang’s model of refugee integration is recognized as a comprehensive framework [18]. Ager and Strang categorized the ten domains of refugee integration into four broad groups (i.e., Foundation, Facilitators, Social Connection, and Markers and Means) [18] as detailed in Table 1.

**Table 1:** Categories and dimensions of Ager and Strang’s integration framework^*^

Category	Dimensions					
Markers and Means	Employment	Housing		Education		Health
Social Connection	Social Bridges (with host community)		Social Bonds (with fellow refugees)		Social Links (with host government)	
Facilitators	Language and Cultural Knowledge			Safety and Stability		
Foundation	Rights and Citizenship					

^*^Adopted from [18]

Within Ager and Strang’s framework, mental health is a key marker and means of integration, reflecting its dual role as a result of and a necessity for successful refugee integration [12]. From a social constructivist view, social issues in health, employment, legal rights, cultural barriers, education, and politics could result in or intensify refugees’ mental problems [19, 20]. Although quantitative studies have highlighted various challenges of integration like status insecurity, family separation, socio-economic pressures, unemployment, and language acquisition affecting refugees’ mental health [21–25], qualitative research could offer deeper insights into refugees’ real-life integration experiences, especially during crises like the COVID-19 pandemic [26, 27].

## Methods

In this study, we employed a qualitative approach to collect and analyze data from U.S. Afghan refugee women and their service providers. Informed consent was obtained from all individual participants included in the study.

**Human Research**: The University of South Carolina’s institutional review board reviewed this study plan under 45 CFR 46.104(d)(2) and 45 CFR 46.111(a)(7) and determined it exempt from human research subject regulations (Pro00121005; 5/16/2022).

### Participants and Recruitment

Participants were recruited from California, which hosts the largest Afghan refugee population in the U.S. [28]. The Sacramento area alone is home to one of the largest Afghan communities in the country after the Taliban takeover, hosting about 40% of new Afghan refugees [29]. Of the Afghan refugee population, women made up 23% and children accounted for 47% [30]. We recruited both Afghan women new to the U.S. post-2021 Taliban takeover and those already long-term U.S. residents.

**Inclusion criteria for Afghan refugee women** were: (1) being an Afghan refugee woman including holders of the Special Immigrant Visa (SIV), (2) having legal U.S. residency, (3) aged between 18–80, (4) speaking Dari, Pashto, or English, (5) having family in Afghanistan, and (6) willing to participate in a one-hour interview. We also recruited refugee service providers for their key role in refugee women’s resettlement [31], insight into refugee integration during the pandemic, and their help in reaching hard-to-reach refugees [32, 33]. **Inclusion criteria for refugee service providers** were (1) being over 18, (2) having at least one year of experience with U.S. refugee organizations, and (3) six months of experience with Afghan refugee women. Participants were recruited using purposive and snowball sampling to ensure diversity in demographics for Afghan women and varied backgrounds for refugee service providers. We followed the concept of information power [34] to ensure a sufficient number of participants were interviewed to provide the depth and breadth of information on research questions (i.e., data saturation). The iterative recruitment and interview process led to rich insights, reaching data saturation with 34 Afghan women and 18 service provider interviews.

### Data Collection

Data was collected through semi-structured interviews over online video platforms like Zoom, WhatsApp, and Skype, as well as face-to-face meetings from August 2022 to July 2023. Open-ended interview questions were designed to elicit participants’ lived experiences of the COVID-19 pandemic and its impact on their integration. Participants were also asked about their demographics. We interviewed participants in Dari, Pashto (using a translator), or English depending on the participant’s choice. Each interviewee provided verbal consent before the interview started. Interviews were audio-recorded, transcribed, and translated into English by the researcher. Afghan refugee women participating in the study received a $50 gift card post-interview to compensate for their time and effort.

### Data Analysis

Analysis of interview data began as early as the first interview was completed using Braun and Clarke’s thematic framework [35] to identify and categorize patterns within data. We employed grounded theory to guide our thematic analysis by critically reviewing interview transcripts to determine proper coding and the formation of themes [36]. The researcher, a female doctoral student in public health, leverages her solid background in health and social sciences for data analysis. Her fluency in Farsi/Dari and working experience with Afghan refugee women provided her with a deep understanding of Afghan women’s culture, religion, and social norms. We used a systematic approach with self-reflection, openness to varying viewpoints, and peer consultations to minimize the researcher’s background influence on the study. The research followed an inductive approach. Using MAXQDA software (version 12, VERBI GmbH, Berlin, Germany), the main themes tied to the primary research questions emerged through systematic code review and were organized into a thematic map. Regular reviews of the transcripts and the summary table were carried out to ensure an across-case and a within-case perspective of context [37]. To address potential biases, input from several Afghan informants was incorporated to finalize the thematic map.

## Results and Discussion

### Descriptive Results

The demographics of the participants in this study are shown in Table 2.

**Table 2:** Demographics of participants

Afghan refugee women (N = 34)		
	**Number**	**Percent**
*Age*		
18–35	21	61.8
36–50	8	23.4
51–65	4	11.8
> 65	1	3
*Occupation*		
Unemployed	26	76.5
Employed	8	23.5
*Marital status*		
Married	22	64.7
Single	4	11.8
Divorced/Widow	8	23.5
*No. of Children*		
0	6	17.6
1–2	12	35.3
3–4	14	41.2
> 4	2	5.9
*English proficiency*		
None	20	58.8
Low	8	23.5
Satisfactory	6	17.7
*Education*		
Elementary and less	8	23.5
Diploma	15	44.1
Associate/Bachelor	10	29.4
Master and above	1	3
*Time of Arrival*		
Before 1/2020	18	53
After 1/2020	16	47
*Immigration Status*		
SIV	16	47.1
Citizen	2	5.9
Refugee	8	23.5
Asylum	6	17.6
Parole	2	5.9
**Refugee service providers (N = 18)**		
*Age*		
18–35	4	22.2
36–50	10	55.6
51–65	4	22.2
> 65	0	0
*Occupation*		
Caseworker	8	44.4
Counselor/therapist	4	22.2
Coordinator/Manager	6	33.4
*Gender*		
Male	4	22.2
Female	14	77.8
*Education*		
Diploma	0	0
Bachelor	8	44.4
Master	7	38.9
MD/PhD	3	16.7

Our study involved 34 Afghan refugee women and 18 refugee service providers. The majority of refugee women were young adults, married, and unemployed. Over half lacked English proficiency with at most a high school diploma. About half of refugee women came to the U.S. before the pandemic. Most refugee service providers were women, occupying various positions such as caseworkers, coordinators or managers, and counselors or therapists. A significant number of service providers held post-graduate degrees. Participants’ demographics in this study reflect the larger Afghan refugee community in the U.S. [28, 38]. Our participants averaged 35.7 years in age similar to the U.S. Afghan immigrant median age of 33 [28]. Participant employment was 23.5%, lower than the 48.7% rate for Afghan refugees in California [38], likely due to gender-based employment gaps [39]. The family dynamics of the participants, predominantly married with 1 to 4 children, resemble the average Afghan refugee household with 4.7 members in the U.S. [38]. English proficiency among the participants was 41.2%, comparable to the proficiency rate (40.8%) among Afghan refugees nationally [38]. The participants’ higher education rate was 32.4%, above the national average (25.8%) for Afghan refugees [38].

### Challenges to the Integration of Afghan Refugee Women in the U.S.

Challenges and facilitators across various socio-ecological levels can influence the integration of Afghan refugee women into U.S. society. Analysis of interview data uncovered challenges and facilitators at all socio-ecological levels, including individual, interpersonal, community, and societal, as detailed below. In the following, we outlined the main challenges to Afghan women’s integration, the COVID-19 impact on these challenges, and their potential indirect effect on integration via mental health impacts.

#### Individual Level

**Increased workload and changes in roles**: Interviews showed increased domestic and financial duties limited Afghan women’s chances for employment and education as key means of integration [18]. The lack of support systems and adjusting to new roles in a different culture hindered their ability to connect socially in their new community. Refugee woman #14 stated, “*Since the time we arrived in the U.S., I have been stressed and pressured. I am a responsible person who has the whole household responsibilities on my shoulders, from state jobs and errands to payments for family education and university tuition fees. However, this time, they are really heavy on my shoulders, and at times, I bend over.*” ***COVID-19 impact*:** The pandemic increased Afghan refugee women’s workload, including more housework, supervising children’s homeschooling, and managing family health, further obstructing their integration into U.S. society.

**A reluctance to engage in work and** over-dependency on aid was indicated by some participants. This reluctance often stemmed from cultural attitudes, lack of confidence, or unfamiliarity with the new environment. Low motivation to work or participate in the community can block economic independence and reduce opportunities for employment and social involvement, key to successful integration [18]. ***COVID-19 impact*:** The rise in government and NGO support during the pandemic and after the 2021 Taliban takeover unintentionally worsened Afghan women’s reliance on these aids. Provider #15 shared, “*Some Afghan women are not willing to work here. I had a case recently where the girl was both a lyricist and journalist but refused to work. I wanted her to join the Afghan Coalition and work voluntarily and then get a position, but she refrains. She just wanted to relax. Once she called and asked for money, then she called again and asked for rent, *etc*.*” ***Mental health***: Over-dependency on aid and lack of self-motivated engagement were accompanied by feelings of helplessness and low self-esteem among Afghan women potentially impacting their integration [40].

**The fear of a new society** stemming from concerns about safety, communication difficulties, and separation from familiar cultural norms, directly impacted the social connection of Afghan women, limiting the development of their social bridges and bonds [18]. This fear also hampered language learning among some Afghan women, crucial for their integration. Provider #9 shared, “*As an Afghan refugee If you have not been assured, you are basically on the streets. In a country, you do not know any language you do not speak. With family, maybe a little baby, that is traumatizing.*” ***COVID-19 impact*:** Pandemic-driven social isolation, health worries, and societal tension escalated fears, further blocking Afghan women’s social connections and cultural navigation in the U.S. ***Mental health***: This fear intensified feelings of helplessness and anxiety due to unfamiliarity with their new surroundings and lack of community support.

**Lack of financial literacy** among Afghan refugee women represented a significant barrier to their integration. Navigating the U.S. financial system is vital for refugee integration, yet the unfamiliarity of Afghan women with complex financial products like credit cards and loans has led to vulnerabilities, such as debt from borrowing without understanding interest rates, impacting their financial stability. This lack of financial literacy not only impeded Afghan women’s economic independence but also affected their ability to secure stable housing. Some women stated challenges in saving for the future, especially for their children’s education. Provider #15 shared, “*To work, they borrowed money to buy a car, and they borrowed money at a high-interest rate because they did not even know what interest rate is. There is no interest rate in Afghanistan as far as my understanding. So, they would be charged with a high-interest rate*.” ***COVID-19 impact*:** The pandemic-induced closure of many NGOs supporting refugees caused a drop in financial education programs for Afghan newcomers, leading to lower financial literacy.

**Age** was another challenge in the integration of Afghan refugee women, especially in the context of COVID-19. Interviews revealed that older Afghan refugee women struggled more with adapting to U.S. cultural norms and learning English, crucial for integration. Their hesitancy to form “social bridges” further isolated them. Older Afghan refugees have faced difficulties in gaining employment due to language barriers, cultural differences, and a lack of understanding of the system. Provider 11 shared, “*In my experience, age greatly affects Afghan women’s integration. For instance, an older Afghan woman I worked with struggled significantly to learn English and adapt to the freedom in American culture. She was reluctant to engage with people outside her family.*” ***COVID-19 impact*:** COVID-19 exacerbated isolation and reduced community participation for older refugees compared to others by amplifying health risks which restricted their involvement in crucial cultural and social activities.

#### Interpersonal Level

A **shift in marital dynamics** after the migration to the U.S. was reported by some women. Refugee woman #14 shared,”*I have a stepsister whose husband just shows up once a week for an hour to pass family expenses and then leaves. In Afghanistan, men were unlikely to have relationships outside of their marriage, but here, due to clubs and other resorts, they have opportunities to establish relationships with other women*.” ***Mental health****:* The disruption of marital stability and trust in their partner has led to a sense of insecurity and vulnerability in some Afghan refugee women which could impact their mental well-being [41].

**Parental challenges**, particularly with infants or children with special needs, have greatly hindered Afghan refugee women’s integration into the U.S. by limiting their employment and education opportunities [18]. This limitation not only affected their economic independence but also restricted their social interactions and ability to engage in community activities, vital for fostering social connections. Refugee woman #5 said, “*After my daughter was born, some problems came up and I could not study my lessons well. I even had issues participating in an online English program. With my kid, having a job was like a dream.*” ***COVID-19 impact*:** The pandemic worsened parental challenges, with school and childcare closures increasing Afghan women’s childcare burden and further isolating them from work and social opportunities. Refugee provider #12 shared, “*I think navigating their children’s lives during the pandemic was really overwhelming for Afghan women and a burden for their adjustment to the U.S.*” ***Mental health****:* These parental challenges escalated the stress level of Afghan women and could lead to a sense of identity loss, leaving them feeling isolated and frustrated [42].

**Isolation** hindered the ability of many Afghan refugee women to establish vital connections within their new environment in the U.S. This challenge significantly affected refugee women’s integration by diminishing their social bridges and bonds [18]. ***COVID-19 impact*:** The pandemic intensified the isolation of Afghan women through increased social distancing and limited direct social opportunities, such as NGO social gatherings and programs shutting down, and the loss of vital social interactions from face-to-face English classes. Refugee woman #25 said, “*I always thought by moving to the U.S., I doomed myself and my children. We had nowhere to go or nobody to see. Here, children have no place to have fun.*” ***Mental health****:* This enforced isolation led to a sense of loneliness and alienation in many Afghan women.

**Domestic/sexual violence** has affected various dimensions of Afghan women’s integration including safety and stability, social bonds, and their rights and citizenship. According to interviews, the experiences of domestic violence by Afghan women hindered their ability to form trusting relationships and feel secure in their new environment. The lack of safety and security experienced in these situations also undermined their ability to exercise their rights and citizenship in the U.S., further marginalizing them. Provider #8 said, “*I recall a DV case where an Afghan woman struggled to seek legal assistance due to the fear of her husband.*” ***COVID-19 impact***: Pandemic lockdowns and resulting stress led to more domestic violence, while the societal shift after the Taliban’s return intensified patriarchal norms among U.S. Afghans, raising domestic and sexual violence risks. Provider #4 said, “*Domestic violence has always been so common among Afghans. However, during the pandemic, the number of DV cases increased significantly. Even these cases are an underestimate of the actual condition of Afghan women since most of them do not report it.*” ***Mental health****:* Exposure to domestic violence resulted in PTSD for some Afghan women, characterized by severe anxiety and nightmares. Some victims also expressed persistent sadness, feelings of hopelessness, depression, and in severe cases, thoughts of suicide.

**Dependency on husbands** impacted the economic independence, social connections, and access to health and education services of many Afghan women. Afghan women’s reliance on their spouses for financial matters, healthcare appointments, and other daily activities restricted their opportunities to engage independently with U.S. society. ***COVID-19 impact***: Pandemic lockdowns and social distancing heightened Afghan refugee women’s reliance on their husbands for outdoor activities, further isolating them and limiting their ability to build external social connections. Refugee woman #28 said, “*I just want to rely on my husband for financial and work matters because I think I do my stuff, I do take care of our home and the children, while my husband is the one who takes care of the money stuff. I do not know about jobs and money here.*”

#### Community Level

**Limited transportation options**: Without a car or knowledge of public transportation systems, Afghan women often struggled to access essential services, engage in employment opportunities, attend their educational programs, or participate in community activities. Furthermore, the absence of safe and dependable transportation options can negatively impact the sense of safety and stability necessary for smooth integration into the new society [18, 43]. Provider #9 stated, “*Another thing is driving, so that these women can at least take their children to different places and help solve some of the problems of the family. They need to be able to work and be involved in social activities and play a role in society.*” ***COVID-19 impact***: The pandemic caused fewer public transportation options and more dependence on personal vehicles, which Afghan women lacked, affecting their access to education, jobs, and healthcare.

**Challenges in housing and resettlement** were reported by some Afghan refugee women, like being relocated multiple times, living in temporary accommodations, and coping with uncertain housing situations. These conditions often forced them into cramped living spaces with extended family, exacerbating stress and limiting their ability to establish a stable home. Moreover, high housing expenses and credit history requirements posed challenges for securing long-term housing. Refugee woman #4 said, “*Since we arrived here, I have been quite stressed due to housing. I needed to live with my brother as well as three children in a small room. After a while, we rented a home. However, rents are so high, and you need to work constantly to meet the expenses.*” These hurdles directly impact refugees’ housing, essential for integration, and also affect their social connections, as stable housing fosters a sense of belonging and community relationships [18]. ***COVID-19 impact***: The COVID-19 pandemic intensified these housing challenges by increasing housing insecurity and restricting the mobility of Afghan women. Moreover, the surge in refugee arrivals following the 2021 Taliban takeover further strained the already limited housing resources.

**Unsafe environments** not only hindered the development of trust and social bridges to the host community for some Afghan women but also disrupted their life stability and potential for establishing lasting relationships. It also impeded their ability to access public services effectively. Refugee woman #31 shared, “*They steal from you in here. When it gets dark, to our horror, we cannot go out. It became worse during the pandemic when most streets were empty. They even point their guns at people, and I witnessed when an armed gang attacked a person.*” ***COVID-19 impact***: The pandemic increased instances of community violence and crime toward refugee women potentially due to the heightened social and economic instability of U.S. society during the pandemic [44]. ***Mental health***: Experiencing violence, harassment, and intimidation led to widespread insecurity among some Afghan refugee women, significantly impacting mental well-being, manifesting as sleep disturbances, hypervigilance, and decision-making difficulties.

#### Societal Level

**Administrative overload**: The interviews highlighted the overwhelming nature of handling state paperwork, navigating bureaucratic processes for essential documents like green cards and passports, and the constant need to manage communications and translations. This administrative load significantly impacts refugee women’s rights and citizenship, heightening uncertainty about securing residency or citizenship essential for integration [18, 45]. Refugee woman #23 shared, “*We have to run and run for every single request like the green card or passport in different organizations. State errands and bureaucracies have driven me crazy and beat.*” Refugee woman #18 shared, “*We took care of one job, while we see our work permit is expired. We then take care of the expired card, then we see there is a problem having just arisen. I got tired of this process and sometimes wished to migrate to another country with less paperwork.*” ***COVID-19 impact***: The pandemic added health and safety administrative tasks, and the 2021 Taliban takeover further complicated legal and immigration procedures, increasing the existing burden. ***Mental health***: The persistent stress and pressure associated with managing a myriad of tasks related to their rights and citizenship led to anxiety and even cognitive overload for some Afghan women.

Language and cultural gaps hinder refugee integration [18], making **religious and cultural disparities** among Afghan refugee women a major barrier to integration in the U.S. Afghan women struggle with balancing cultural preservation and adaptation, as they navigate between their traditional norms and the liberal social environment in the U.S., leading to internal conflicts and societal pressures, especially among the younger generation. An aged refugee woman (#11) said, “*I dislike the freedom running in here, because we are Moslems, and these indecent scenes are not pleasing to us at all*.” The efforts to maintain cultural and religious values while adapting to new surroundings caused isolation and misunderstandings for some women, weakening their social bonds and bridges and hindering their integration. Limited knowledge of U.S. culture has also impeded their integration [18]. Service provider #12 stated, “*We had a recent event. Some were in favor of music while some were against playing music. They were very happy about one night you know that was in there, but overall provided us some cultural issues.*” ***Mental health***: Cultural and religious conflicts cause stress and alienation in Afghan refugee women, worsening their well-being. Discrimination and bullying over cultural practices also intensified feelings of isolation and depression among these women.

**Language barriers** also challenged the integration of Afghan refugee women in the U.S. Language, a primary behavioral indicator of acculturation [46], has a key role in the integration of refugees by affecting employment, education, housing, and health as the main means of integration [18]. The language skill gap in Afghan women impeded their effective communication in the U.S., limiting their participation in employment and education, and hindering connections within their community and with government services. Provider #10 said, “*The biggest barrier to the integration of many Afghan women is English. They cannot communicate without language. They cannot speak when they go to school or the doctor. They even cannot get a simple without knowing English.*” ***COVID-19 impact***: The pandemic exacerbated this issue by reducing opportunities for in-person language learning and practice for Afghan refugee women. ***Mental health***: Limited language skills have caused isolation, frustration, and helplessness among some Afghan refugee women, especially when facing critical situations like health issues or legal matters.

Interviews showed that Afghan women, previously in respected roles in Afghanistan, often experience a **loss of social status** in the U.S., taking jobs well below their qualifications. This challenge can hinder Afghan women’s integration by limiting their access to employment and education, with further negative effects on housing and social welfare. ***COVID-19 impact***: The pandemic has exacerbated this challenge by escalating employment difficulties and limited opportunities, intensifying the loss of social status among Afghan women. ***Mental health***: This loss of social class has led to feelings of devaluation and disrespect as well as frustration, loss of identity, and low self-esteem in some Afghan women which could affect their mental well-being [47].

**Financial hardship** was another barrier to the integration of Afghan refugee women in the U.S. Financial strain, high living costs, and scarce job opportunities have pushed refugee women into low-paying, unstable employment, affecting their ability to secure stable housing crucial for integration. ***COVID-19 impact***: COVID-19 intensified the financial hardships of Afghan refugee women by increasing unemployment and reducing work hours. ***Mental health***: Many Afghan women, used to Afghanistan’s economic structure, struggle with the financial demands in the U.S., increasing their anxiety and sense of helplessness. Refugee woman #27 shared, “*In Afghanistan, I did not need money. I was a teacher and had an income. Even if I needed it my family was always there to help. However, I feel I financially need way much more here as I am both unemployed and more in need. Here, we have no money to shop and buy something for our children.*”

**Patriarchal culture/restriction on Afghan women** was among the top challenges to the integration of Afghan refugee women in the U.S. Cultural norms often restrict Afghan women’s autonomy and decision-making, as some cannot work or leave the house without their husband’s permission, limiting their economic independence. Husbands controlling their wives’ clothing choices or attendance at counseling not only restricts Afghan women’s access to social and professional opportunities but also reinforces gender inequality and perpetuates dependency and disempowerment. Patriarchal culture impedes Afghan women’s ability to form social bonds and access rights, directly affecting their social connections, and rights and citizenship [18]. Provider #11 shared, “*Afghan men are frequently afraid of having women’s rights respected in here and women are entitled to do what they are willing to. That is why as soon as Afghan men enter the U.S. and see the conditions, they are willing to control their wives the same way they did in Afghanistan. They want to keep everything under their supervision and control, things like going out, shopping, visiting around, hitting parties, *etc*. everything about women must be under their supervision.*” ***COVID-19 impact***: Both pandemic-induced home confinement and the Taliban’s takeover intensified patriarchal controls of Afghan women in the U.S., further limiting their mobility and resource access, and severely undermining their integration. ***Mental health***: The clash between Afghan women’s desires for autonomy and restrictive family and community expectations led to stress, anxiety, and a sense of powerlessness for some women, impacting their mental well-being.

**Limited recognition of Afghan educational degrees** posed another challenge to the integration of Afghan refugee women in the U.S. This barrier limits Afghan women’s education and employment, crucial for integration and economic independence. It also limited their potential social interactions at the workplace with the U.S. community, further hindering their social bridges. ***COVID-19 impact***: The pandemic’s economic downturn and increased job competition have exacerbated the employment struggles of Afghan women with unrecognized degrees. Refugee woman #4 shared, “*I attended college and studied midwifery for two years in Afghanistan. I earned my diploma. However, when we arrived here during the pandemic, my diploma was not evaluated as a U.S. equivalent degree. That is why I have never had a job here in my field.*”

Some Afghan women have expressed concerns about **stigma** as a barrier to their personal growth (e.g., learning English, education) in the U.S., which in turn has affected their ability to integrate effectively. Some women did not participate in or drop out of English or computer classes just because they were worried about being judged or ridiculed. Refugee woman #30 stated, “*I am really worried about being ridiculed by others in the classes I take because I do not know anything. I am illiterate.*” ***Mental health***: Many refugee women avoided counseling in the U.S. due to the stigma and labeling associated with therapy in Afghan culture, negatively impacting their mental well-being. Refugee woman #33 said, “*I am worried they might call me insane for going to a counselor.*”

**Limited access to healthcare** posed another barrier to the integration of Afghan refugee women in the U.S. ***COVID-19 impact***: The pandemic severely restricted Afghan women’s access to medical services, notably impacting pregnant women. It hindered their integration process by limiting their social connections, community participation, and employment stability. Refugee woman #20 said, “*Having access to healthcare has always been one of our issues, especially in the COVID-19. We always had challenges with making appointments with doctors, earning a referral to a medical expert, and conducting costly experiments. My sister was pregnant during the pandemic and got infected too. Doctors arranged visits for months later which is crazy.*”

The **U.S. job market** presented another barrier to the integration of Afghan refugee women. The challenge lies in the non-transferability of Afghan women’s skills and experiences from Afghanistan to the U.S. context, particularly in understanding local systems and laws. Additionally, a lack of familiarity with job-seeking processes in the U.S., such as CV writing and interview preparation, further complicated their employment. ***COVID-19 impact***: The economic downturn and increased competition in the job market during the pandemic have made it more challenging for Afghan women to find suitable jobs. Provider #5 shared, “*Getting a job here is so difficult for Afghan women because if you had a full career in Afghanistan, it does not transit to the U.S. It is different here. Some of them did not even know English well enough to know how to look for a job and land one.*”

**Inefficient policies/programs** was another barrier to the integration of Afghan refugee women in the U.S. Interviews revealed that Afghan refugees receive insufficient orientation and support after the initial six months of resettlement, with programs failing to adequately prepare them for broader social and life integration in America, leaving many feeling overwhelmed and unprepared. Provider 4 stated, “*There should have been more steps planned for refugees. There should have been a lot more policy written for after leaving a military base. And there should be more orientation. There is a huge learning process that has to happen about how you make it in America, you know, instead of just like okay there you go, goes as your welcome money and figure it out.*” ***COVID-19 impact***: Pandemic-driven NGO closures and reduced programs limited the essential support and orientation needed for the effective integration of Afghan women into U.S. society. ***Mental health***: The sense of being unprepared and lacking support eroded the confidence of some Afghan women in their capacity to thrive in their new surroundings, increasing their feelings of despair and depression.

### Facilitators of the Integration of Afghan Refugee Women in the U.S.

#### Individual Level

**Empowering Afghan refugee women** in the U.S. was a significant facilitator of their integration. This empowerment has encompassed various dimensions, including education, employment, and personal development. For example, Women for Afghan Women (WAW) provides legal assistance, immigration support, and counseling to Afghan women facing domestic violence or legal vulnerability. Afghan Coalition offers microenterprise training and job-readiness workshops to support women in building economic independence. It also hosts health education, civic participation, and family support programs tailored to Afghan cultural needs. International Rescue Committee (IRC) implements the Afghan Youth Success Program, empowering young Afghan women with mentoring, leadership training, and life skills. They offer ESL, financial literacy, and job placement services to adult women. ***COVID-19 impact***: The pandemic, however, posed challenges to these empowerment efforts. Firstly, Afghan women should enhance their language abilities and education to adapt to new environments, but the pandemic hindered these efforts by shifting to less effective online methods or halting them entirely. Secondly, Afghan women have struggled with cultural and logistical employment barriers like childcare or driving skills, worsened during the pandemic as job openings dwindled and the shift to remote work proved difficult for those unfamiliar with digital technology or lacking home-based work options. Lastly, personal development is crucial for Afghan women’s empowerment, but the pandemic limited their opportunities to engage in community events, access support services, and communicate with American neighbors, impeding their empowerment and integration. Provider #5 shared, “*I think the problems these women are grappling with are due to their low literacy. They do not know English and never drive, which is why they are restricted. As they are jobless, they are always tense and stressed. If they can go out like everybody, have a job like everybody, and get to sort out their problems, I think women then can be satisfied with themselves and their families.*”

**Positive orientation to the future** was another facilitator to the integration of Afghan refugee women in the U.S. This positive outlook, often expressed by newcomers as hope and determination to build a better life, empowered Afghan refugee women to pursue employment, education, and health care, all of which are essential markers and means of their integration process [18]. Refugee woman # 2 shared, “*I always say: this is exactly the right place for you, so you should shake a leg, learn something, and earn a bachelor’s degree. I should sooner or later study and keep my education up. This is the only thing encouraging me so much.*” A forward-looking perspective helped Afghan women build social connections within the U.S. community and their peers, fostering meaningful relationships and motivating them to learn new skills, thereby bridging cultural and language gaps. Additionally, this mindset contributed to a sense of stability, providing the psychological resilience needed to navigate the complexities of resettlement and integration in the U.S. ***COVID-19 impact***: The pandemic has caused extensive interruptions in employment, education, and healthcare services for Afghan refugee women, sparking doubts among some of them about the prospects of a promising future. ***Mental health***: A positive orientation towards the future contributed to the improved resilience of Afghan women, enabling them to cope with the stress of resettlement.

**Technological skills** can facilitate the integration of Afghan refugee women into the U.S. The ability to use social media platforms like Facebook and TikTok enabled some Afghan women to stay connected with their community, supervise their children’s online activities, and engage with broader society. These skills enhanced their social bonds, social bridges, and understanding of new culture, key elements in refugees’ integration. ***COVID-19 impact***: The pandemic increased the need for online communication and remote interaction skills to maintain social ties in isolating times and strengthen refugee women’s connections with the U.S. government. ***Mental health***: Afghan women with advanced technological skills experienced better mental well-being during the pandemic due to being less isolated. Refugee woman #25 said, “*I am a member of TikTok, I have created an account to be in contact with my daughter during the COVID-19, mostly to supervise her and know what she is doing. I used it a lot and I am in touch with my other Afghan friends and people from work.*”

#### Interpersonal Level

**Support from family/friends/community** was a significant facilitator to the integration of some Afghan refugee women in the U.S. This support provided Afghan women with crucial information, emotional support, and technical help which facilitated their access to jobs and housing, expanded their cultural understanding, and helped them navigate and adapt to their new environment. For instance, a refugee woman (#4) highlighted how crucial a friend’s information about state benefits for Afghans was in securing stable housing upon arrival. Provider #1 stated, “*I think having a sponsored family is a strong facilitator for their engagement in the U.S. community. They took care of them in a kind and patient way and helped them to be part of this community. We had several positive and successful experiences for refugees having a sponsored family.*” However, not all Afghan women had positive experiences; some noted a lack of assistance from relatives in America who failed to provide guidance or help in obtaining necessary support and resources. The ethnic difference was a major barrier for Afghan women to receive support from their community which was exacerbated after the 2021 Taliban takeover. Provider #18 said, “*Another unique and predominant feature among Afghans is their multiplicity in their tribal origin and viewpoints, despite that they all came from the same country. For example, some fellows cannot get on with people from another tribe: A Kazak fellow cannot bear a Tajik guy, or a Tajik fellow never puts up with a Pashto fellow, or a Pashto dude finds it impossible to stand another guy from another tribe, and the loop goes on.*” Furthermore, the community can sometimes enforce restrictive norms, especially around cultural and gender roles. This indicates a negative social capital for Afghan refugee women manifested through restrictive social norms and obligations imposed by their community, which may hinder their progress and adaptation in the host society [48]. This form of social capital can lead to exclusion from broader societal resources and opportunities, ultimately affecting Afghan refugee women’s integration and well-being. These differences can also be explained by the concept of “Fragmented Ties” introduced by Cecilia Menjívar which argues how adverse immigration policies, limited economic prospects, and resource-scarce communities could lead to conditional and inconsistent support among immigrants, challenging the norm that networks are reliable sources of assistance, especially in contexts where poverty hinders mutual aid [49]. ***COVID-19 impact***: The necessity for social distancing in the pandemic limited physical gatherings and reduced the opportunities for Afghan refugee women to receive face-to-face support, guidance, and information sharing from their support networks. ***Mental health***: Emotional support from family and friends helped alleviate feelings of isolation and loneliness for some Afghan women. Yet, for others, the lack of support, particularly from relatives already established in America, heightened feelings of stress and abandonment.

#### Community Level

**Socializing** was another facilitator of the integration of Afghan refugee women in the U.S. Social gatherings have facilitated Afghan refugee women’s integration by providing cultural exchange, emotional support, skill-building opportunities, and enhancing connections within both their Afghan and U.S. communities, sometimes even leading to new job opportunities. Provider #2 shared, “*I think social gatherings for Afghan women should be more. I mean we should have a place where Afghan women come and talk and learn some skills. I want them to have a network. These actions would help Afghan women to become independent and successful here.*” ***COVID-19 impact***: During the pandemic, restrictions on social gatherings reduced the effectiveness of support groups and social activities, but virtual platforms partially compensated for it by maintaining social connections. ***Mental health***: Socializing reduced feelings of isolation among Afghan women by connecting them with others facing similar experiences, fostering a sense of community and belonging.

**Childcare support** emerged as a vital facilitator for the integration of Afghan women with children into the U.S., significantly influencing their social and personal well-being. Within Ager and Strang’s integration framework, childcare support could affect employment, education, health, and social connections crucial for refugee integration. Although scarce, the childcare programs available to Afghan women enabled them to participate in English and job skills development programs and boosted their employment opportunities, health management, and social involvement by freeing up time for self-care. Some providers noted childcare support as a critical facilitator for many NGO services to Afghan women. Provider #9 said, “*It would be so good if they provided some childcare support programs so that Afghan women could participate in the educational classes and English programs offered to them.*” Moreover, educating Afghan mothers on child nutrition has equipped them with the knowledge to enhance their children’s diets and health, which is essential for their development and future engagement in work and community life. ***COVID-19 impact***: The pandemic heightened the importance of childcare support for Afghan women’s integration, as increased home confinement and closures of childcare facilities led to reduced social connections and limited participation in employment or educational activities.

**Access to special needs services**, like Individualized Education Programs (IEP) for children with autism, significantly aided the integration of Afghan mothers with special needs children. An Afghan refugee mother (#27) shared, “*I do appreciate the State for the priceless support they did to me and my daughter. She is in the process of IEP, which has been offered for free. They relieved much of my stress here.*” ***COVID-19 impact***: The pandemic hindered refugee women’s access to these services, causing delays and cancellations of in-person sessions.

#### Societal Level

A**wareness of laws and women’s rights** in the U.S. could be a powerful facilitator for the integration of Afghan refugee women, as it empowers them to defend their rights and navigate the new societal norms. This knowledge was vital for overcoming cultural barriers, enabling many Afghan women to form social connections and understand legal rights, providing a foundation for their safety, stability, and integration into American society. Provider #12 shared, “*We need to work on the contents that motivate women to defend their rights and never accept what their husbands impose. We need to let them know they have the same rights as their husband. Like their husband, they can have their credit, their finances.*” ***COVID-19 impact***: The pandemic restricted Afghan refugee women’s social interactions and educational opportunities, as social distancing measures curtailed in-person gatherings and workshops, limiting their chances to learn about their rights and legal protections in the U.S. Refugee woman #24, a newcomer, said, “*I do not know anything about the U.S. laws or our rights here*.”

Providing Afghan women with **culturally competent jobs** was a significant facilitator in the integration of Afghan refugee women. Jobs tailored to the cultural strengths and needs of Afghan women enhance their integration by providing culturally competent and accessible employment, such as home-based roles or jobs in female-dominated workplaces, helping them balance work and family responsibilities while fostering skill development and financial independence. Provider #6 shared, “*You should get them a job to do at home. In the course I took, many Afghan women were happy to be working from home. I remember some of them sewed. Therefore, you ought to support them by getting them a sewing machine so that they can sew*.” Culturally competent jobs can bridge cultural gaps by utilizing Afghan women’s native skills or fostering interactions within their community, enhancing their sense of belonging and well-being; however, interviews indicate these opportunities are limited in the U.S. ***COVID-19 impact***: While the pandemic increased unemployment and reduced job opportunities for Afghan women, the shift to remote work and the growing need for flexible employment expanded the availability of culturally competent jobs for them.

**Freedom in the U.S.** also played a role in facilitating the integration of Afghan refugee women. The freedoms in the U.S., including gender equality and education rights, significantly advanced the social integration and personal development of Afghan women, empowering them towards economic independence and self-sufficiency, and positively impacting their rights, safety, stability, and education. Refugee woman #18 shared, “*I have gone through a big change since I arrived in the U.S. My outlook toward life has changed. In Afghanistan, women just kept living on their basic needs, but here, they get to know that they are humans and have equal rights as men in society. Both women and children enjoy humane values here.*” ***COVID-19 impact***: COVID-19 has hindered the benefits of U.S. freedoms for Afghan women by limiting their social interactions and available opportunities for their empowerment.

### Subgroup Analysis of Refugee Integration

The interviews highlighted numerous challenges and facilitators of integration that predominantly impacted married Afghan women with children. Challenges such as heightened workload, altered roles, shifts in marital dynamics, parenting difficulties, domestic violence, being dependent on husbands, and patriarchal culture were commonly noted by these women. On the other hand, the adverse effects of the COVID-19 pandemic on integration facilitators, such as women’s empowerment, childcare support, access to special needs services, and knowledge of legal rights and women’s rights, were frequently mentioned by married Afghan mothers. Single mothers frequently cited financial difficulties as a significant obstacle to integration. Educated Afghan women reported that the loss of social status and the limited acknowledgment of their academic qualifications were major barriers to their integration. An overreliance on assistance was commonly seen among single and young Afghan women. Additionally, service providers noted that Afghan women with illiteracy and low English proficiency were more vulnerable to domestic and sexual violence.

### Conceptual Model of Afghan Refugee Women’s Integration in the U.S.

**Fig. 1:**
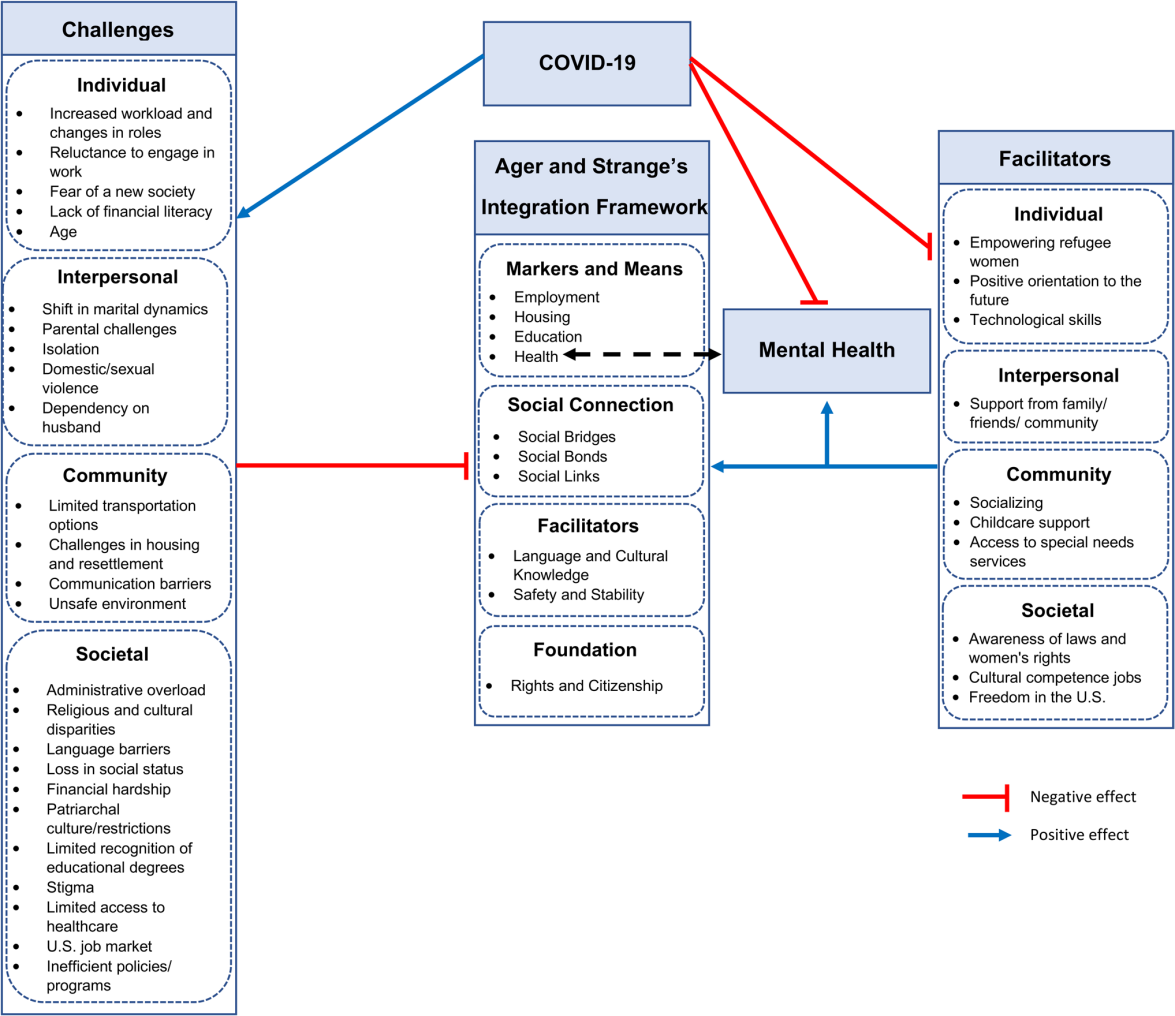
The conceptual model of U.S. Afghan refugee women’s integration in the COVID-19 pandemic context

Drawing from the challenges and facilitators of the integration of Afghan refugee women uncovered in the interviews, we developed a conceptual model tailored to integration for Afghan refugee women living in the U.S. in the COVID-19 context, as illustrated in Fig. 1. Challenges and facilitators were organized according to the socioecological theory into individual, interpersonal, community, and societal levels.

### Public Health Policies and Programs

According to our findings, the integration of Afghan refugee women into U.S. society is a complex layered process that requires multifaceted policies and programs addressing various socio-ecological levels.

#### Individual and Interpersonal Levels

***Enhanced Support Services***: Develop and expand support services that specifically address the increased workload, financial literacy, and psychological stress experienced by Afghan refugee women. These services might include mental health support, financial literacy programs, and family counseling to address the shifts in marital dynamics and parenting challenges exacerbated by the COVID-19 pandemic. ***Empowerment Programs***: Implement empowerment and personal development programs to boost Afghan women’s confidence and independence. Programs aimed at improving language skills, job readiness, and digital literacy can empower women to pursue employment and education more vigorously.

#### Community Level: Community Integration Programs

Foster community engagement opportunities that reduce isolation and build social connections. These programs can include community centers offering language and cultural exchange programs, social gatherings, and skill-building workshops tailored to the needs of Afghan women. ***Transportation and Childcare Support***: Enhance access to essential services by providing transportation solutions and expanding childcare support. These measures will facilitate Afghan women’s participation in the workforce and educational programs, addressing key barriers to their integration.

#### Societal Level: Policy Reforms for Inclusive Integration

Advocate for policy reforms that recognize the unique challenges faced by Afghan refugee women, including the non-recognition of educational credentials and the need for gender-specific integration policies. Policies should aim to facilitate smoother transitions into the job market and educational institutions for Afghan women. ***Public Awareness and Anti-Discrimination Campaigns***: Implement public awareness campaigns to combat stigma, discrimination, and cultural misunderstandings. These campaigns can promote a more inclusive society that values diversity and supports the rights and integration of refugee women. ***Legal and Citizenship Support***: Expand legal and citizenship support services to address the administrative overload and uncertainties faced by Afghan women. Streamlining bureaucratic processes and providing clear, accessible information on legal rights and citizenship can alleviate stress and promote a sense of security among Afghan refugees. ***Culturally Competent Job Opportunities***: Promote the creation of culturally competent job opportunities that leverage the skills, experiences, and cultural backgrounds of Afghan women. Partnerships with local businesses and organizations can help create these jobs. ***Healthcare Access and Education***: Improve healthcare access for Afghan women through targeted outreach programs and education on navigating the U.S. health system. Special attention should be given to overcoming language barriers and providing culturally competent health services.

### The Dual Realities of Afghan Refugee Women Integrating into U.S. Society

The coexistence of struggle and optimism observed among Afghan refugee women in this study reflects the complex and often contradictory nature of forced migration and experiences of refugees [50]. While interviews with Afghan women highlighted profound structural, cultural, and psychosocial barriers—including poverty, isolation, and gender-based restrictions—many women also expressed future-oriented resilience and a desire to rebuild their lives through education and employment. This dichotomy may illustrate the dynamic process of adaptation, where hardship does not preclude hope [51]. For example, the concept of "freedom" emerged as both a source of empowerment and discomfort: while some participants viewed American freedoms (e.g., gender equality and individual rights) as enabling personal growth, others associated certain expressions—such as public displays of affection—with cultural dissonance or moral decline. It shows that refugee integration is not a linear path, but rather an ongoing negotiation between preservation of cultural identity and selective adoption of new norms [12]. While refugees often face cumulative adversity before and after migration, the development of a hopeful disposition enables them to reframe their experiences and pursue long-term goals, making hope both a coping mechanism and a pathway toward empowerment and successful adaptation [51].

## Conclusion

In this study, we investigated the multifaceted and layered process of integration for Afghan refugee women into U.S. society during the COVID-19 pandemic. Through conducting interviews with Afghan refugee women and their service providers, we identified the challenges and facilitators of the integration of Afghan refugee women across various socio-ecological levels (i.e., individual, interpersonal, community, and societal). We assessed the impact of the COVID-19 pandemic on these factors and their potential implications for Afghan refugee women’s mental health. The study revealed numerous challenges to the integration of Afghan refugee women, exacerbated during the pandemic, from increased workloads and financial illiteracy to the challenges of adapting to a new culture and navigating the complex U.S. job market and health systems. Empowerment through education and employment, community support and engagement, and access to culturally competent job opportunities, emerged as critical facilitators in supporting the integration of Afghan refugee women into American society. This study emphasizes the critical need for public health policies and programs tailored to the integration challenges and facilitators of Afghan refugee women across various socio-ecological levels, to support their integration which has been impaired by the pandemic.

## Data Availability

Data is provided within the manuscript or supplementary information files.
